# Plasma ProBNP Is Not a Specific Marker for Transient Myocardial Ischemia

**DOI:** 10.14740/jocmr2024w

**Published:** 2015-05-08

**Authors:** Khawar Maqsood, Muhammad T. Shakoor, James R. Cook, Gregory R. Giugliano, Amir Lotfi

**Affiliations:** a Department of Cardiology, Baystate Medical Center, Tufts University School of Medicine, Springfield, MA, USA; b Department of Medicine, Baystate Medical Center, Tufts University School of Medicine, Springfield, MA, USA

**Keywords:** ProBNP, Acute myocardial ischemia

## Abstract

**Background:**

Plasma proBNP levels are increased in patients with acute myocardial infarction. Previous studies have shown conflicting data on the effect of transient myocardial ischemia on plasma BNP levels. We designed the current study to examine plasma proBNP levels in patients with transient myocardial ischemia during a percutaneous coronary intervention (PCI). This study was to study plasma proBNP as a marker of transient myocardial ischemia.

**Methods:**

We enrolled 49 consecutive patients with a history of angina or abnormal stress test who presented for cardiac catheterization. We obtained plasma proBNP levels in all patients at 1) arterial access (proBNP-1), 2) the end of the procedure (proBNP-2) and 3) 4 hours after procedure (proBNP-3). Hotelling’s T-squared test was used to evaluate the equality of means. Log transforms of proBNP were used to impart data normality.

**Results:**

Twenty-two patients underwent diagnostic catheterization (DCA group) and 27 underwent PCI (PCI group). Both groups had normal left ventricular function and a baseline creatinine < 2 mg/dL. Baseline log (proBNP) was 4.7 + 0.99 (units) and rose significantly at 4 hours in both groups (P < 0.02), with no difference in rate of change.

**Conclusions:**

Plasma proBNP was increased in both DCA and PCI groups which limits its utility to identify transient myocardial ischemia. The etiology of increase in proBNP in both groups is speculative and may be related to injection of radiographic contrast media into the coronary artery which leads to microcirculatory impairment resulting in myocardial tissue hypoxia and transient increase in left ventricular pressure; however, further evaluation is required.

## Introduction

Brain natriuretic peptide (BNP) was originally discovered in porcine brain extract and later in human brain and ventricular myocardium. Studies have indicated that the natriuretic peptides (atrial natriuretic peptide, BNP and C-type natriuretic peptide) have actions which include natriuresis, vasodilatation, inhibition of the renin-angiotensin-aldosterone axis, and inhibition of the sympathetic nerve activity [1]. Elevation of plasma BNP and N-terminal proBNP (proBNP) has been documented in patients with congestive heart failure (CHF), asymptomatic left ventricular (LV) systolic dysfunction, LV hypertrophy and diastolic dysfunction [2-7].

Plasma levels of BNP and proBNP have also been shown to increase after acute coronary syndrome and have prognostic implications [8-12]. Plasma BNP level increases after acute myocardial infarction and the rise is directly proportional to the infarct size and inversely proportional to the post-infarction ejection fraction [13, 14]. Kikuta et al reported a high BNP level in patients with unstable angina which decreased towards normal after treatment [15]. The stimulus for BNP release is myocyte stretch [16] but there is evidence that ischemia can lead to increased expression of BNP mRNA in hypoxic ventricular muscle through hypoxia-inducible factor-1 transcription factor [17]. Studies have shown that in patients with coronary artery disease (CAD) and normal LV systolic function, BNP rises abruptly after exercise and the rise is proportional to the size of ischemic territory [18, 19]. Contrary to these findings, there have been studies showing no clinical significance of BNP in acute ischemia and CAD [20-22]. We sought to investigate the association between transient myocardial ischemia induced by balloon inflation during PCI and plasma concentrations of proBNP amongst patients undergoing diagnostic cardiac catheterization and PCI.

## Methods

### Study population

The study was approved by the Baystate Medical Center Institutional Review Board. We enrolled 49 consecutive patients with a history of angina or abnormal stress test who presented to Baystate Medical Center for cardiac catheterization. Twenty-two patients underwent coronary angiography with PCI (PCI group) and 27 patients underwent diagnostic coronary angiography (DCA group).

### Blood sampling

We obtained plasma proBNP levels in all patients at 1) the initial arterial access (proBNP-1), 2) the end of the procedure (proBNP-2) and 3) 4 h after procedure (proBNP-3). We used Roche Diagnostics Corporation (Indianapolis, IN) assay for proBNP measurement with either serum or heparinized plasma using the Elecsys 1010/2010 and the Modular Analytics E170 immunoassay analyzers.

### Exclusion criteria

Patients with a history of CHF, severe pulmonary disease, renal insufficiency (serum creatinine > 2.0 mg/dL), left bundle branch block, permanent pacemaker, valvular heart disease, unstable angina, non-ST-elevation myocardial infarction (NSTEMI), and previous LV systolic dysfunction were excluded.

### Statistical analysis

Baseline patient data are presented as mean ± SD or proportions where appropriate. The Wilcoxon signed rank test was used to evaluate the equality of means and Hotelling’s T-squared test was performed to assess the equality of mean vectors over time. Since the proBNP data were positively skewed, log transforms of proBNP were used to impart data normality when necessary. Statistical significance was determined at a P-value of less than 0.05.

## Results

**Figure 1:**
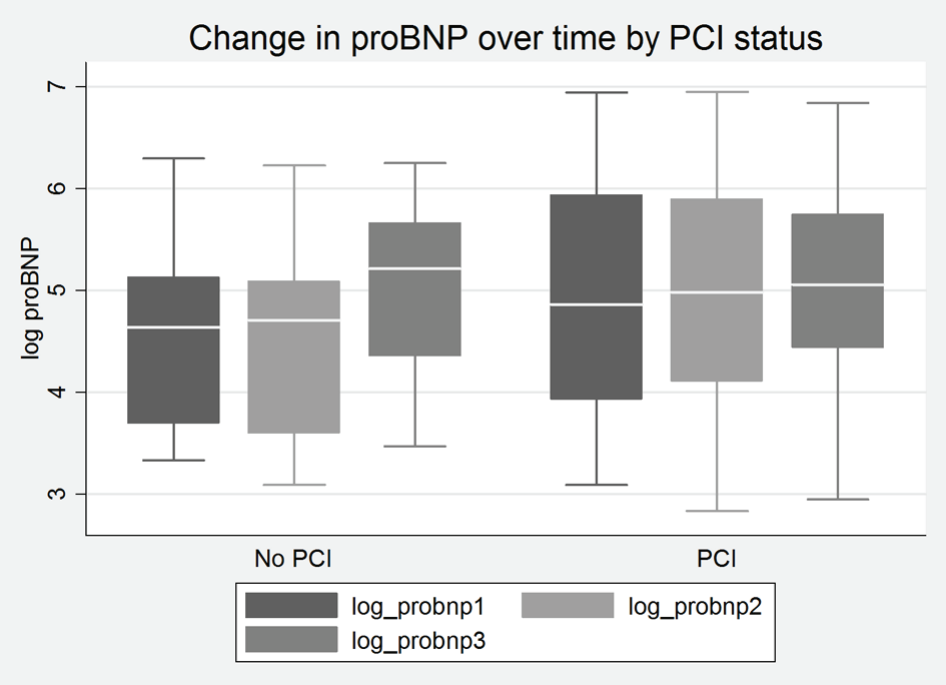
Change in proBNP level over time in PCI and DCA (no PCI) groups. Log values are used to impart data normality.

Baseline demographic, medication profiles, and procedural characteristics were well matched between the groups (Table 1). As expected, contrast dose was significantly higher in the PCI group (234 ± 12.4 cc vs. 132 ± 43.4, P = 0.003). Although the LVEF in both groups was within the normal range, the LVEF was significantly higher in the DCA group (P = 0.04). However, there was no systolic dysfunction or evidence of clinical CHF in either group. There was a non-significant rise in proBNP-2 and proBNP-3 levels in both groups (Fig. 1). There was no difference in LVEDP between the two groups. Mean proBNP-1, proBNP-2 and proBNP-3 in the DCA group and the PCI group are shown in Table 2. As noted, baseline proBNP levels were non-significantly higher in the PCI group. In the DCA group, there was no association between proBNP levels and severity of CAD. No significant correlates with proBNP were found amongst the baseline characteristics listed in Table 1 including gender.

**Table 1:** Baseline Characteristics

Characteristics	DCA group	PCI group	P value
Number of patients (n = 49)	22	27	NS
Age (mean ± SD)	60 ± 11.1	61 ± 12.5	NS
Gender (% female)	45	33	NS
LV EF % (mean ± SD)	61.3 ± 7.7	57 ± 5.6	0.04
LVEDP (mean ± SD)	16 ± 5.2	18 ± 5.6	NS
Hypertension (%)	63.6	66.6	NS
Diabetes mellitus (%)	22.7	14.8	NS
Hyperlipidemia	59	59.2	NS
Active smokers (%)	4	11	NS
BUN (mean ± SD)	18.4 ± 5.2	15.8 ± 6.3	NS
Serum creatinine (mean ± SD)	0.94 ± 0.22	0.94 ± 0.3	NS
Beta-blocker (%)	72	77	NS
ACE inhibitor/ARB (%)	31.8	48.1	NS
Statin (%)	59	59.2	NS
Diuretics (%)	22.7	29.6	NS
Contrast (mean ± SD)	132 cc ± 43.4	234 cc ± 124.8	0.003

NS: non-significant; DCA: diagnostic coronary angiography; PCI: percutaneous coronary intervention.

**Table 2:** Mean ProBNP-1, ProBNP-2 and ProBNP-3 Values in DCA and PCI Groups

	Mean proBNP-1 (pg/mL)	Mean proBNP-2 (pg/mL)	Mean proBNP-3 (pg/mL)	P value
DCA group	131.15 ± 122.17	129.13 ± 114.76	198.9 ± 136.67	NS
PCI group	225.17 ± 258.3	223.34 ± 250	241.5 ± 234.1	NS

## Discussion

Multiple studies have demonstrated the utility of plasma proBNP and BNP levels in the diagnosis of CHF and in prognostication of patients with heart failure and acute coronary syndrome [2, 5-12, 23, 24]. There are limited data on the value of BNP and proBNP in the setting of acute myocardial ischemia. Tatieshi et al investigated the release of BNP in patients undergoing diagnostic angiography and percutaneous transluminal coronary angioplasty (PTCA). They demonstrated that plasma BNP levels peaked at 24 h only in the PTCA group but did not change significantly in patients undergoing diagnostic catheterization. No significant changes were seen in the hemodynamic parameters immediately before and 24 h after PTCA. No relationship was seen between duration of angioplasty balloon inflation and plasma BNP elevation [25]. Kyriakides et al, in a small study (n = 13), studied ANP and BNP levels in patients with stable angina and normal LV function undergoing PTCA. They found that BNP levels peaked at the end of angioplasty and returned to baseline at 4 h. They proposed that ischemia induced rise in LVEDP may lead to rise in BNP [26]. Karla et al studied 28 patients with stable angina and elevated baseline proBNP levels that underwent single vessel angioplasty with serial plasma proBNP measurements. They demonstrated a significant decrease in plasma proBNP levels at 24 h post-intervention compared to baseline levels. They speculated that the potential reasons for decrease in proBNP level may be due to decrease in ischemic burden or changes in LV function [27]. McClure et al studied proBNP levels in 26 patients with LAD stenosis and normal baseline LV function undergoing PCI. They showed a significant decrease in proBNP levels 8 weeks after PCI. They proposed that diastolic dysfunction secondary to ischemia can be the potential reason for high proBNP levels in patients with obstructive CAD [28].

Our study demonstrated that plasma proBNP levels were not significantly associated with transient ischemia induced by cardiac catheterization or during PCI. However, it is consistent with Goetze et al study demonstrating coronary angiography induces a transient increase in proBNP values [29]. Our results are different from previous studies conducted by Tatieshi et al and Kyriakides et al which showed a significant rise in BNP levels in patients undergoing PTCA. The non-significant rise in proBNP in the PCI group of the present study can be explained by previous studies suggesting a strong correlation between higher BNP values with significant obstructive CAD and extent of CAD [30-32]. Ischemia has been shown to lead to increased BNP mRNA expression [17] and in experimental acute myocardial infarction, BNP synthesis is increased in infarcted as well as non-infarcted myocardium [33]. In previous studies, the rise in BNP after PTCA and exercise was early and rapid which points towards release of stored BNP rather than *de novo* synthesis of BNP after acute ischemia [12]. BNP has certain important actions including inhibition of the renin-angiotensin-aldosterone axis, inhibition of the sympathetic nerve activity [1] and vasodilation in coronary vasculature [34]. These attributes and the fact that BNP rises abruptly with exercise in patients with known CAD [18, 19] point towards a counter-regulatory effect of BNP against ischemic insult and the higher levels seen in patients with obstructive CAD are likely to be compensatory. The reason for increase in proBNP following DCA is speculative but may be related to injection of radiographic contrast media into the coronary artery which leads to microcirculatory impairment resulting in myocardial tissue hypoxia and transient increase in LVEDP [35]. During coronary angiography the exchange of blood with contrast media solution causes periods of hypoxia and during ventriculography both ionic and non-ionic monomeric contrast media cause a decrease sinus oxygen difference with a tendency to increase myocardial oxygen consumption [36]. However, our results do not support this correlation. We recognize that the present study is limited by the small sample size which limits the power to detect associations that were tested.

### Conclusion

In summary proBNP, although helpful in diagnosis of heart failure and many other conditions [37-39], does not appear to have a strong association with myocardial ischemia. Based on our findings we conclude that proBNP should not be used for diagnosis of acute myocardial ischemia. Further studies are needed to evaluate the role of proBNP, if any, in the setting of acute myocardial ischemia.
